# A rat model of a focal mosaic expression of PCDH19 replicates human brain developmental abnormalities and behaviours

**DOI:** 10.1093/braincomms/fcac091

**Published:** 2022-04-05

**Authors:** Andrzej W. Cwetsch, Ilias Ziogas, Roberto Narducci, Annalisa Savardi, Maria Bolla, Bruno Pinto, Laura E. Perlini, Silvia Bassani, Maria Passafaro, Laura Cancedda

**Affiliations:** Brain Development and Disease Laboratory, Istituto Italiano di Tecnologia, Via Morego 30, Genova 16163, Italy; Università degli Studi di Genova, Via Balbi 5, Genova 16126, Italy; Instituto de Biotecnologia y Biomedicina (BIOTECMED), Universidad de Valencia, Burjassot 46100, Spain; Brain Development and Disease Laboratory, Istituto Italiano di Tecnologia, Via Morego 30, Genova 16163, Italy; Università degli Studi di Genova, Via Balbi 5, Genova 16126, Italy; Brain Development and Disease Laboratory, Istituto Italiano di Tecnologia, Via Morego 30, Genova 16163, Italy; Brain Development and Disease Laboratory, Istituto Italiano di Tecnologia, Via Morego 30, Genova 16163, Italy; Dulbecco Telethon Institute, Rome, Italy; Brain Development and Disease Laboratory, Istituto Italiano di Tecnologia, Via Morego 30, Genova 16163, Italy; Università degli Studi di Genova, Via Balbi 5, Genova 16126, Italy; Brain Development and Disease Laboratory, Istituto Italiano di Tecnologia, Via Morego 30, Genova 16163, Italy; Bio@SNS, Scuola Normale Superiore, Piazza dei Cavalieri 7, Pisa 56126, Italy; Brain Development and Disease Laboratory, Istituto Italiano di Tecnologia, Via Morego 30, Genova 16163, Italy; CNR Institute of Neuroscience, Milan 20129, Italy; CNR Institute of Neuroscience, Milan 20129, Italy; Brain Development and Disease Laboratory, Istituto Italiano di Tecnologia, Via Morego 30, Genova 16163, Italy; Dulbecco Telethon Institute, Rome, Italy

**Keywords:** PCDH19-CE, mosaicism, epilepsy, autism, *in utero* electroporation

## Abstract

Protocadherin 19 gene-related epilepsy or protocadherin 19 clustering epilepsy is an infantile-onset epilepsy syndrome characterized by psychiatric (including autism-related), sensory, and cognitive impairment of varying degrees. Protocadherin 19 clustering epilepsy is caused by X-linked protocadherin 19 protein loss of function. Due to random X-chromosome inactivation, protocadherin 19 clustering epilepsy-affected females present a mosaic population of healthy and protocadherin 19-mutant cells. Unfortunately, to date, no current mouse model can fully recapitulate both the brain histological and behavioural deficits present in people with protocadherin 19 clustering epilepsy. Thus, the search for a proper understanding of the disease and possible future treatment is hampered. By inducing a focal mosaicism of protocadherin 19 expression using *in utero* electroporation in rats, we found here that protocadherin 19 signalling in specific brain areas is implicated in neuronal migration, heat-induced epileptic seizures, core/comorbid behaviours related to autism and cognitive function.

## Teaser

Cwetsch *et al*. report a rat model of focal brain mosaicism of PCDH19 downregulation induced by *in utero* electroporation, aimed at mimicking the X-linked inheritance pattern of PCDH19-CE people. A local mosaic of Pcdh19 downregulation resulted in brain development deficits, heat-induced epileptic seizures, autism-related behaviors, and reduced cognitive performance.

## Graphical Abstract

**Graphical Abstract:**
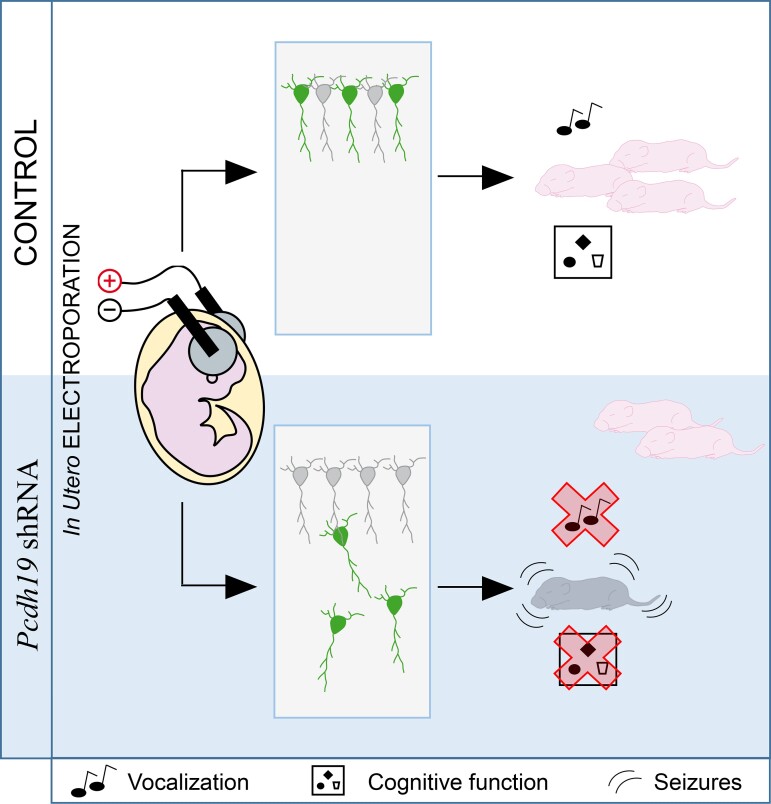

## Introduction

Mutations in the protocadherin 19 (*PCDH19*) gene located on chromosome X (Xp22.1) cause female-limited epilepsy [*PCDH19* gene-related epilepsy or PCDH19 clustering epilepsy (PCDH19-CE); OMIM #300088]. PCDH19-CE is an infantile-onset epilepsy syndrome also characterized by psychiatric (including autism-related), sensory, and cognitive impairments of varying degrees.^1,2,3,4,5,6^ PCDH19 is part of a large family of cell-adhesion molecules that play specific roles in the patterning and wiring of diverse brain areas during development. Accordingly, PCDH19-CE people also present brain structural abnormalities. In particular, together with areas of normal architecture, focal dysplasia, heterotopia, and abnormal morphology of individual neurons in the cortex as well as hippocampal sclerosis have been reported.^7,8,9,10,11,12,13^ Due to random X-chromosome inactivation, PCDH19-CE-affected females are composed of a mosaic population of healthy and *PCDH19*-mutant cells, whereas hemizygous male carriers are asymptomatic or show much reduced psychiatric and behavioural deficits.^3,13,14,15^ To explain sex differences, a cellular interference model has been proposed. According to this model, random X inactivation in females leads to tissue mosaicism in which cells expressing the wild-type (WT) PCDH19 protein and cells expressing a mutant PCDH19 protein coexist, and thus scramble cell–cell communication and integration in neuronal circuits.^7,15,2,16^ Thus, cellular interference between two populations of cells (i.e. WT and PCDH19-mutated cells) is a possible cause of brain dysfunctional development, leading to symptoms in PCDH19-CE people.^17^ Accordingly, the rare cases of fully affected males that have been described arise from somatic mutations that display mosaicism.^13,15,16,18,19,20^

In this light, an ideal model of choice for PCDH19-CE studies appears to require a cellular interference strategy. Recently, a number of animal models have been generated to identify the molecular mechanism underlying PCDH19-CE pathophysiology. Although these animal models are being instrumental and have started to shed light on the histopathology of the disease, they do not fully recapitulate the PCDH19-CE phenotype regarding overt brain malformations together with significant behavioural deficits. This would be a pre-requisite for future testing of potential therapeutic treatments. Moreover, while characterized by global mosaicism in all brain areas, the current models (although very valuable) do not allow to dissect which particular affected brain area is responsible for the diverse behavioural phenotypes.^8,11,21,22,23,24,25,26^ Herein, we used *in utero* electroporation (IUE) to achieve a focal mosaic of PCDH19-downregulated cells intermixed with WT cells in the rat cortex or hippocampus, and thus mimicked the focal disorganization of the brain tissues described in PCDH19-CE-affected people. By performing histological and behavioural studies in female and male animals, we found that PCDH19 signalling in specific brain areas is implicated in neuronal migration, heat-induced epileptic seizures, core/comorbid behaviours related to autism and cognitive function.

## Materials and methods

### Animals

All animal procedures were approved by the Istituto Italiano di Tecnologia (IIT) licensing in compliance with the Italian Ministry of Health (D. Lgs 26/2014) and EU guidelines (Directive 2010/63/EU). Sprague–Dawley (SD) rats were housed in filtered cages in a temperature-controlled room with a 12:12 h dark/light cycle and *ad libitum* access to water and food. We used male and female rats in all experiments. The total amount of animals used in this study is summarized in Table 1. Distribution of the animals for the specific experimental procedures is summarized in Table 2.

**Table 1:** Numbers of animals used each particular experiment/figure

Figure	Number of processed animals	
	Control	*Pcdh19* shRNA
1A and B	3 per time point	N/A
1C–F	1	N/A
2C	7	–
2D	5	–
2E and G	3	3
2F and H	3	3
3A and B	7	6
3C and D	5	6
4B	7	9
4C and D	7	6
4E–H	34	26
4I	17	18
4J and K	6	7
5A and B	9	7
5C–E	3	7
6C	12	17
6E	7	8

**Table 2:** Cohorts and ages of the animals used in the behavioural test

Animals	Epileptic Events	Vocalization	Huddling	Hot plate	Social behaviours	Novel object recognition	Fear conditioning	Histology
Cohort 1	P7							
Cohort 2		P9						P9
Cohort 3			P9	P14				P25
Cohort 4					P38–39			
Cohort 5						P25		P25
Cohort 6							P28–P35	

### Western blot

Rat cortices were dissected in cold phosphate-buffered saline (PBS) on ice and lysed immediately in lysis buffer [2% sodium dodecyl sulphate (SDS), 2 mM ethylenediaminetetraacetic acid (EDTA), 10 mM 4-(2-hydroxyethyl)-1-piperazineethanesulfonic acid, pH 7.4, 150 mM NaCl with 1 mM PMSF, 10 mM sodium flouride, 1% protease and phosphatase inhibitor cocktails (Sigma)]. Lysates were then sonicated and clarified by centrifugation (15 min at 20 000×*g*). For immunoblot analysis, equal amounts of protein estimated with a bicinchoninic acid assay (BCA) kit (Pierce) were run on 10% polyacrylamide NuPAGE precast gels (Invitrogen), subjected to electrophoresis, and transferred onto nitrocellulose membranes (GE Healthcare). Membranes were probed with primary antibodies against actin (1:5000, Sigma) and PCDH19 (1:1000, Novus), followed by peroxidase-conjugated anti-rabbit (1:5000, BioRad) or anti-mouse (1:5000, BioRad) secondary antibodies. Stained membranes were developed with SuperSignal West Pico chemoluminescent substrate, and bands were quantified by measuring the mean intensity signal using ImageJ. Images of stained membranes available in Supplementary Fig. 1.

### Generation of constructs

short hairpin RNA (shRNA)#1 was a gift from the Maria Passafaro laboratory, Istituto di Neuroscienze del CNR, Milan.^36^ For shRNA#2, a 21-nucleotide target sequence was chosen with the aid of BLOCK-iT^™^ RNAi Express Software (Invitrogen). shRNA #1 and 2 target sequences were chosen from different regions of the mRNA. Their specificity for the mRNA of interest was verified by BLAST aligning with the NR database. shRNAs were synthetized and cloned into the pLVTH vector expressing green fluorescent protein (GFP); shRNA#1: 5′-GAGCAGCATGACCAATACAAT-3′; shRNA#2: 5′-GCTTCTGCCCTTGTCCTAA-3′. As a control, we used scrambled shRNAs with the following sequences: 5′-GCTGAGCGAAGGAGAGAT-3′ and 5′-GCCCATCCTTCGCGTTATT-3′ for shRNA#1 and shRNA#2, respectively. shRNA#1 was validated in the study by Bassani *et al*.^36^ shRNA#2 was co-transfected with a construct expressing rat PCDH19 in COS7 cells, and the expression of PCDH19 was assessed by western blot (data not shown: empty vector: 82.89 ± 9.1%; scrambled shRNA: 100 ± 5.45%; shRNA: 9.48 ± 2.34%). shRNA#2 significantly downregulated PCDH19 expression (one-way ANOVA; *post hoc* Holm-Sidak: **P < 0.01).

### *In utero* electroporation

Animal care and experimental procedures were conducted in accordance with the IIT licensing and the Italian Ministry of Health. Surgeries were performed following published protocols for the laboratory.^27,28^ E17.5 timed-pregnant Sprague–Dawley rats (Harlan Italy SRL, Correzzana, Italy) were anaesthetized with isoflurane (induction, 3.5%; surgery, 2.5%), and the uterine horns were exposed by laparotomy. The mix of shRNA#1 and shRNA#2 (1:1) or corresponding control scrambled shRNAs (4–6 µg/µl in water) plus pCAGGs IRES GFP (0.5 µg/µl) and the dye Fast Green (0.3 mg/ml; Sigma) was injected (5–6 µl) through the uterine wall unilaterally. For huddling test, control animals were electroporated with pCAGG IRES GFP and shRNA scrambled vector; PCDH19-downregulated animals were electroporated with pCAGG IRES Td-Tomato and the mix of shRNA#1 and shRNA#2. This allowed easier identification (and huddling experiment design) of the treated versus control animals, just by looking at the green versus red fluorescence under a fluorescent lamp, respectively. After a single hemisphere injection, the embryo’s head was placed between tweezer-type circular electrodes [10 mm, somatosensory cortex (SSC) electroporation] or tweezer-type circular electrodes (10 mm) and a third additional electrode (5 mm × 6 mm, hippocampus electroporation). For the electroporation protocol, we applied five electrical pulses (amplitude, 50 V; duration, 50 ms; intervals, 150 ms) by a square-wave electroporation generator (ECM 830 device; BTX, Massachusetts, USA). After electroporation, the uterine horns were returned into the dam’s abdominal cavity, and embryos allowed continuing their normal development. In the study, only one hemisphere was electroporated. Left and right hemispheres were at times electroporated with control vectors and at times with Pcdh19 shRNA, to avoid biases. For animals electroporated with the experimental *Pcdh19* shRNA, littermates electroporated with control plasmids were used in the same behavioural sessions as controls.

### Histology and immunostaining

Brains were fixed by transcardial perfusion with 4% paraformaldehyde in PBS, cryopreserved in 30% sucrose, frozen and sectioned coronally (80 µm thickness) using a microtome with a freezing unit (Microm HM 450 Sliding Microtome, Thermo Scientific). Free-floating slices were permeabilized and blocked with 0.3% Triton X-100 and 10% normal goat serum (NGS) in PBS. Brain slices were incubated with the primary antibodies anti-PCDH19 (Rabbit, 1:500, Novus), anti-NeuN (Rabbit, 1:500, Cell Signaling Technology) or anti-GFP (Mouse, 1:600, AbCam or Chicken, 1:600, AbCam) in 0.3% Triton X-100 and 5% NGS in PBS overnight at 4°C. Immunostaining was detected using fluorescent secondary antibody anti-mouse (Alexa 488, 1:600, Thermo Fisher) or anti-rabbit (Alexa 568 and 647, 1:600, Thermo Fisher) in PBS containing 0.3% Triton X-100 and 5% NGS for 2 h at room temperature. Slices were counterstained with Hoechst (1:1000 Sigma). Samples were mounted in Vectashield Mounting Medium (Vector Laboratories, Burlingame, CA, USA) and processed for confocal microscopy. We used GFP staining on electroporated slices to increase the GFP signal intensity for better visualization of transfected cells. Td-Tomato-fluorescent intensity was already strong enough, and did not require enhancement by immunohistochemistry. This was possibly due to the fact that GFP was expressed driven by the U6 promoter in the shRNA vector, and Td-Tomato was expressed driven by modified chicken β-actin promoter with a cytomegalovirus-immediate early enhancer in the pCAGGS vector, which confers high and long-lasting *in vivo* expression.

### Confocal acquisition and analysis

For the quantification of IUE-density of the SSC (Layer II/III) and hippocampus (CA1 region), images from 80-µm-thick sections counterstained with Hoechst and NeuN were acquired on a confocal laser-scanning microscope (TCS SP5; Leica Microsystems, Milan, Italy) equipped with a 63× immersion objective [numerical aperture (NA): 1.4; 2 µm-thick z-stacks]. Three z-stacks were projected on a 2D image, and Hoechst- or NeuN-positive cells were manually counted using the ‘Cell Counter’ plugin of Fiji. For each image, the electroporation density was estimated by calculating the percentage of Td-Tomato cells over either Hoechst^+^ or NeuN^+^ cells. For each slice, two random fields in the central part of the electroporated region were acquired, and their density values were averaged. One or two slices were analyzed for each animal. For IUE specificity and migration analysis, images from brain sections counterstained with Hoechst were acquired on a confocal laser-scanning microscope equipped with a 10× immersion objective (NA: 0.3). Confocal images (15-µm-thick z-stacks) were acquired, and z-series were projected into two-dimensional (2D) representations. For the quantification of electroporation specificity as well as non-migrating cells, all cells in somatosensory Layer II/III or hippocampal CA1 region were counted and normalized to the total number of fluorescent cells in the slice. For spine counting, confocal images were acquired using a confocal laser-scanning microscope equipped with a 63× immersion objective (NA 1.4) with 1.5× digital zoom (0.5-µM-thick z-stacks) and pixel size: 0.16 µm. Acquired stacks (three images) were projected on a 2D image. The images were not de-convoluted. On each image, basal dendrites of a randomly chosen transfected neuron were visually identified, and spines were manually counted (using the ‘Cell Counter’ plugin of Fiji^29^) for the whole visible length of one to three secondary dendrites and divided by their respective length. Spine densities obtained for each dendrite were then averaged for each neuron. One or two images for one to three different slices were acquired per animal.

### Ultrasonic vocalization test

All pups tested for ultrasonic vocalization (USV) were first electroporated *in utero* at E17.5 with control or *Pcdh19* shRNA. Electroporated pups at P9 were separated from their mother and littermates and placed one by one in an empty container (diameter, 5 cm; height, 3 cm). The empty container was then placed in a sound-attenuating Styrofoam box (diameter, 30 cm; height 40 cm). Five-minute recordings of USVs were performed with a microphone sensitive to frequencies of 10–180 kHz ultrasound (Avisoft UltraSoundGate condenser microphone capsule CM16, Avisoft Bioacoustics, Berlin, Germany) and Avisoft Recorder software. The number of calls was calculated using Avisoft SASLab Pro.

### Hyperthermia-induced seizure test

Experimental procedures were performed following a protocol previously described^30^ with small adjustments. P7 rat pups were placed in a Plexiglas cylinder (12 × 15) heated by a heat lamp (red light Philips IR 250 RH IR2 230–250 V 250W. LUX: 1.000–1.200 Lux) positioned approximately 50 cm above the cylinder. The pup temperature was monitored both by an external thermal probe connected to a temperature control unit (Temperature Control Unit HB 101/2, Panlab, Harvard Apparatus) and by an infrared temperature gun. Temperature was elevated by approximately 0.5°C every 1.5 min (∼0 min), until reaching hyperthermia (temperature >39°C) and maintained between 39 and 41.5°C for 20 min. Following the hyperthermia protocol, pups were rehydrated by subcutaneous saline injection, cooled on a metal surface and returned to their dams. The total separation time from the dam was maintained <40 min. The experiment was video-recorded and the duration of epileptic events defined as shaking, falling, tail shaking, myoclonic jerks, limb clonus, and generalized tonic-clonic epilepsy^30^ were measured manually by a blind operator.

### Huddling test

Experimental procedures were performed as previously described by Naskar *et al*.^31^ with a modification in quantifications. In particular, each litter was formed by 10 pups with a proportion of control versus *Pcdh19* shRNA animals set to be strictly to 5:5. Control versus *Pcdh19* shRNA pups at the first days after birth were identified via a fluorescent lamp. This allowed visualization of (transfected) fluorescent cells through the pup scull, which has no fur at that developmental stage), of in^32^ Red (*Pcdh19* shRNA) versus Green (control) fluorescence identification was used to arrange the experimental groups for the huddling. For the rest of the huddling experiment and all other behavioural experiments, behavioural assessments were performed by an operator ‘blind’ to the phenotype, and the codes were broken only after brain histological assessment and behavioural data quantification. At P9, littermates were isolated from their mother and introduced in an empty arena (50 cm × 50 cm) where they were all separated from one another by ∼9.4 cm on the circumference of a circle (30 cm diameter) drawn on the floor of the arena. Ten-minute videos (camera: Canon XF105 HD Camcorder, Canon; Sony HXRNC2500 AVCHD Camcorder, Sony) of freely moving pups were recorded. From the recordings, we extracted one frame every 30 s and visually scored the behaviour of all pups in huddling groups based on their proximity and interaction. We considered a pup doing huddling when it made active and prolonged contact with one or more littermates by using his head, snout, or when it formed a pile with them. A custom Python script (Naskar *et al*.)^31^ was used to extract from the scores four different descriptive parameters: Time Spent Together (the time that each pup spent forming a cluster with every other littermate) Time Spent Isolated (the time that each pup spent outside of a cluster).

### Hot plate test

The response to an acute thermal stimulus was measured in pups at P14 using an adapted hot plate test.^33,34^ The experimenter gently placed the pup on the surface of the hot plate kept at 52°C. The latency to withdraw the paw from the hot plate was measured. To prevent any heat injury to pups, a cutoff latency of 30 s was applied.

### Three-chamber test

The test evaluates the social approach of the test rat versus a novel animal (Stimulus 1) in comparison with an object (sociability) or versus a second novel intruder animal (Stimulus 2) in comparison with Stimulus 1 (social novelty). This test was performed in a three-chambered box [apparatus comprises a rectangle, three-chambered box of gray acrylic, evenly illuminated by overhead red light (12–14 lux)] placed in a dark room, as previously described.^35^ In the apparatus, the three chambers are accessible by rectangle openings with sliding doors. In the first 10 min of the test (habituation), the tested rat was free to explore the apparatus with two empty rat cages (one in each of the two side chambers), with a cone-shaped lid to prevent the rat climbing on the top of the cages. Then, the tested rat was briefly confined in the central chamber while the Stimulus 1 (previously habituated to the apparatus) was placed inside a cage placed in one of the side chambers. For the following 10 min (sociability test), the tested rat was allowed to explore all three chambers. Then, the tested rat was again briefly confined in the central chamber while the Stimulus 2 (previously habituated to the apparatus) was placed in the other side chamber inside an empty cage. For the following 10 min (social novelty test), the tested rat was allowed to explore all the three chambers. The time spent exploring the object or the stimulus (interaction time) was calculated by measuring the number of seconds the rat spent showing investigative behaviour (i.e. head orientation, sniffing occurring within <1.0 cm from the cages). In addition to the interaction time, we also calculated the time spent in the chamber, where the object or the stimulus were placed. The Sociability Index for the interaction time was calculated as the difference between the time spent investigating the Stimulus 1 (T1) and the time spent interacting with the familiar object (T0) divided by the total exploration time: the Sociability Index for the interaction time = (T1 – T0)/(T1 + T0). The Social Novelty Index for the interaction time was calculated as the difference between the time spent interacting with the Stimulus 2 (T2) and the time spent interacting with the Stimulus 1 (T1) divided by the total exploration time: Social Novelty Index for the interaction time = (T2 – T1)/(T2 + T1). Both the Sociability and Social Novelty Indexes were also calculated for the time spent inside the chambers.

### Novel Object Recognition test

The Novel Object Recognition (NOR) test was conducted in a gray acrylic arena (44 cm × 44 cm). On Day 1, the rat was allowed to habituate to the apparatus by freely exploring the arena for 15 min. On Day 2, during the acquisition sessions, three different objects were placed into the arena, and the rat was allowed to explore for 15 min. Object preference was evaluated during the sessions. Twenty-four hours after the acquisition session, the rat was placed in the same arena with one object replaced by a novel object and was allowed to explore freely for 15 min. We considered exploratory behaviour to be direct contact with the object. In case of indirect or accidental contact with the objects, the event was not included in the scoring. The time spent exploring each object was expressed as a percentage of the total exploration time for each trial. The discrimination index was calculated as the difference between the time spent investigating the novel object (*T*_n_) and the time spent investigating the familiar objects (*T*_f_) over the total amount of exploration time of the novel and familiar objects: Discrimination Index = (*T*_n_ – *T*_f_)/(*T*_n_ + *T*_f_). The test was performed under infrared illumination. The test was performed under infrared illumination.

### Contextual Fear-Conditioning test

Each rat was individually moved from its home cage to the fear-conditioning system (TSE Systems) consisting of a transparent acrylic conditioning chamber (44 cm × 44 cm) equipped with a stainless-steel grid floor. After 3 min, the rat received one electric shock (constant electric current: 2 s, 1.5 mA) through the floor. Fifteen seconds after the shock, the rat was removed from the apparatus and placed again in its home cage. On the next day, the rat was placed in the same conditioning chamber for 3 min (trained context), and 2 h later, it was moved to a novel environment (black chamber with gray plastic floor and vanilla odour, untrained context) for 3 min. The freezing behaviour observed in the trained and untrained context was scored and normalized to the total time spent in the chamber. The test was performed under infrared illumination.

### Statistical analysis

Statistical analysis was performed with Prism 7 (GraphPad Software) and R (R Core Team, version 3.6) software. All of the data are presented as the mean scores ± SEM. Equal distributions of the variances and normal distributions of the residues were inspected by Levene’s and Shapiro–Wilk tests, respectively; if one or more violations of parametric test assumptions were detected, the corresponding non-parametric test was run. Specifically, instead of the standard *t*-test, the Mann–Whitney test was performed when the normality assumption or both assumptions were not met; the Welch’s *t*-test was employed when only the assumption of equality of variances was violated. The Holm method was used to correct for multiple comparisons.

## Results

### PCDH19 expression is temporally and spatially regulated in the rat brain during development

**Figure 1:**
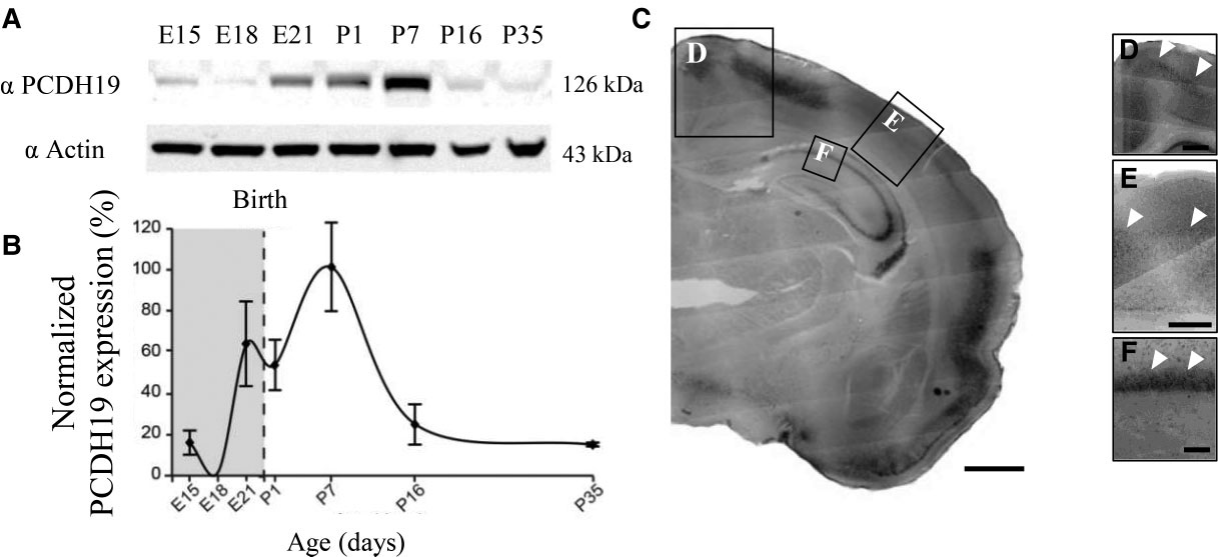
**PCDH19 is highly expressed during perinatal cortical development**. (**A**) Representative western blot showing the temporal expression of PCDH19 in comparison with actin in lysates of rat brain cortices at different ages. (**B**) Quantification of PCDH19 average expression (±SEM) in experiments as in (**A**). PCDH19 signal was normalized to actin levels for each age. Data are shown as percentage of peak PCDH19 expression normalized to P7 levels. *n* = 3 animals for each age. (**C**) Representative anti-PCDH19 staining of a coronal section of a rat brain at P7, showing localization of PCDH19-positive cells in the motor, SSc, and hippocampal SP. Scale bar: 200 µm. (**D–F**) Higher magnification images (as in the area indicated with squared frames on the right) of the motor cortex, SSc and CA1 hippocampal region. White arrows point to high expression of PCDH19. Scale bar: 50 µm.

We previously demonstrated that PCDH19 shows a gradual increase in expression from E18 to P7, followed by a successive decline in the rat hippocampus.^36^ Here, we investigated the expression of PCDH19 during rat cortical development by western blot analysis. PCDH19 was poorly expressed at embryonic stages, increased during postnatal (P) development to reach a peak at P7, and subsequently decreased later in development and early adulthood (Fig. 1A and B). Next, to investigate the spatial expression of PCDH19 at its peak (P7), we immunostained coronal brain sections and detected PCDH19 levels in the cortex and hippocampus (Fig. 1C). We found that PCDH19 showed a very widespread pattern of expression in the motor cortex (Fig. 1D), whereas in the SSc its expression was predominantly in Layer IV (Fig. 1E). Moreover, PCDH19 was highly expressed in the hippocampus, especially in the stratum pyramidale (SP; Fig. 1F). Altogether, these results indicate that PCDH19 is temporally and spatially expressed at diverse levels in different brain areas.

### Targeted IUE leads to region-specific mosaicism

**Figure 2:**
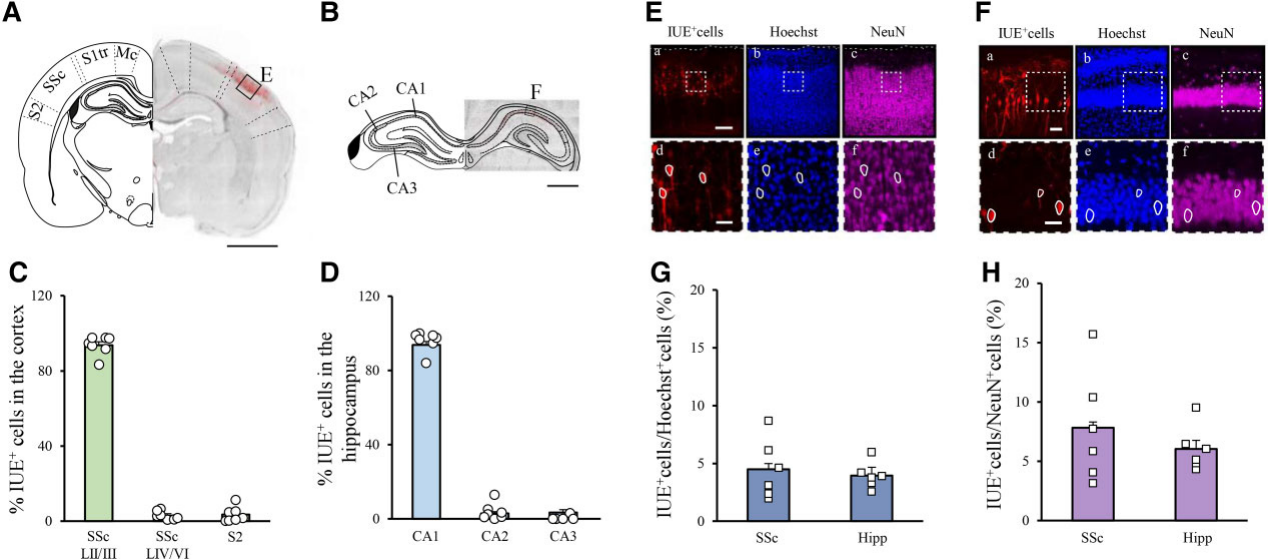
**IUE of E17.5 rat embryos can specifically target Layer II/III neurons of the SSc or CA1 neurons of the hippocampus**. (**A**) Example of a brain coronal slice of the SSc of a P9 pup previously electroporated at E17.5 (**B**) Example of a brain coronal slice of the hippocampus from a P9 pup previously electroporated at E17.5. Slices were counterstained for Hoechst. Electroporated (IUE^+^) cells expressed the reporter Td-Tomato. Scale bars: 2 mm (**A**), 1 mm (**B**). Bregma (±): −2.92. The left hemisphere was replaced by a cartoon map [modified from Paxinos and Watson (1997)], highlighting the diverse cortical areas (**A**): motor cortex (MC), SSc—trunk region—(S1tr), SSc, secondary SSc (S2), or hippocampal areas (B): cornu ammonis 1 (CA1), 2 (CA2) and 3 (CA3). (**C**) Quantification of the IUE specificity for the SSc, Layer II/III, SSc layers IV–VI, and S2, expressed as a number of GFP^+^ cells in that region divided by the total amount of GFP^+^ cells in the electroporated section. One–two slices averaged for each animal; total number of animals used *n* = 7. (**D**) Quantification of the IUE specificity for the CA1, CA1, and CA3 hippocampal regions expressed as a number of GFP^+^ cells in that region divided by the total amount of GFP^+^ cells in the electroporated section. One section for each animal; total number of animals used *N* = 5. (**E, F**) High magnification of the square highlighted in (**A**) and (**B**), respectively, showing IUE^+^ Td-Tomato-expressing cells (a), Hoechst^+^ cells (b) and NeuN^+^ neurons (c) are shown for the same acquisition field. Scale bars 150 µm (**E**), 50 µm (**F**). (d)–(f): further magnifications of white-dashed squares in (a)–(c), employed for calculating the IUE-density; the contour of some electroporated cells is shown in white to facilitate the visualization of the overlap between the different markers. Scale bars: 30 µm (**E**), 30 µm (**F**). (**G, H**) Quantification of the average percentage of IUE^+^ cells over Hoechst^+^ (**G**) and NeuN^+^ cells (**H**) for the Layer II/III of the SSc and the CA1 region of the hippocampus, as indicated in (**A**) and (**B**), respectively. Squares indicate values from single animals (two fields per slice and two slices averaged for each animal; total number of animals used *N* = 6), and their averages (±SEM) are reported as bars.

To mimic the mosaic expression of PCDH19 in PCDH19-CE, we reproduced a model of focal mosaicism by using the IUE technique to target a subpopulation of neuronal progenitors of the cortex or hippocampus in rat embryos at embryonic day (E)17.5. By this means, after neuronal differentiation and completion of the neuronal migration process to the cortex or hippocampus, we obtained brain area-specific cell subpopulations of transfected neurons interspersed with untransfected neurons. To assess the specificity of our electroporation protocol, we first electroporated *in utero* a plasmid encoding the fluorescent reporter protein Td-Tomato in a configuration designed to target either the SSc or the CA1 region of the hippocampus, and assessed the number of transfected cells in pups at postnatal day (P)9 in each brain region. We found that IUE specifically targeted the desired Layer II/III of the SSc or the hippocampus CA1, as quantified by the number of Td-Tomato-positive cells (Td-Tomato^+^) over the total number of Td-Tomato^+^-transfected cells in the slice from each specific brain region (SSc Layer II–III pyramidal neurons: 93.6 ± 1.93%; CA1 hippocampal region 95.9 ± 2.1%, Fig. 2A–D). Next, to quantify the density of transfected versus untransfected cells in the SSc or the hippocampal CA1 region, we performed immunostaining for the cell marker Hoechst and the neuronal marker NeuN in the brain slices from P9 rat pups previously electroporated *in utero* with Td-Tomato. When we calculated the percentages of electroporated (Td-Tomato^+^) cells over total (Hoechst^+^) cells for Layer II/III of the SSc (Fig. 2E) and for the CA1 region of the hippocampus (Fig. 2F), we found approximately 5 and 4% of total number of neurons in the selected region, respectively (Fig. 2G). This percentage increased to 8% for the SSc and 6% for the hippocampus when we normalized the count to NeuN^+^ neurons (Fig. 2H).

### PCDH19 downregulation in the SSc causes neuronal migration deficits, increased seizure susceptibility, and core/comorbid behaviours related to ASD

**Figure 3:**
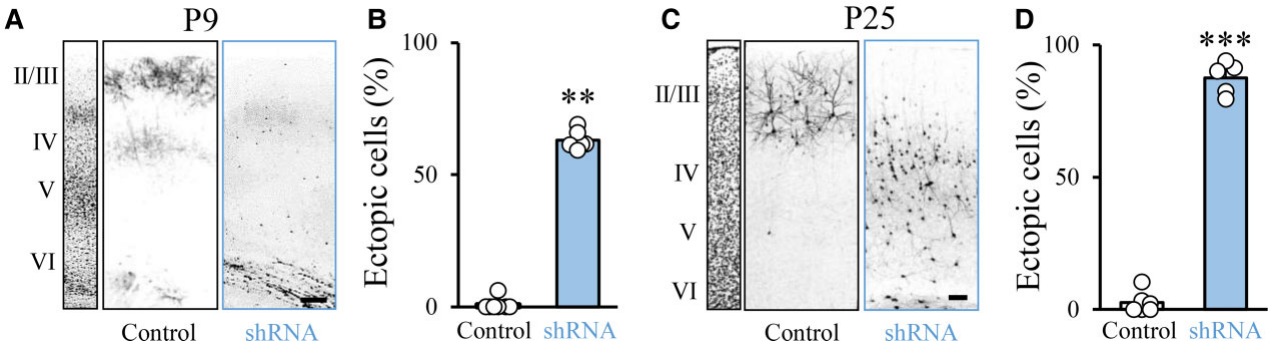
**Mosaic PCDH19 downregulation in the SSc impairs neuronal migration**. (**A, C**) Confocal images of GFP fluorescence in coronal sections of rat SScs at P9 and P25 after IUE at E17.5 with control vector or functional *Pcdh19* shRNA. Slices were counterstained with Hoechst for visualization of cortical layers (left). Scale bar, 50 µm (**A**) and 100 µm (**C**). (**B, D**) Quantification of the number of ectopic cells transfected with either control vector or *Pcdh19* shRNA against *Pcdh19*. Numbers are expressed as the average percentage of ectopic cells normalized to the total number of fluorescent cells in the same section (± SEM). Mann–Whitney test: **P = 0.002 (**B**), Student’s *t*-test: ***P < 0.0001 (**D**). Total number of animals processed (one slice/animal): (**B**) control: 7; *Pcdh19* shRNA: 6; (**D**) control: 5; *Pcdh19* shRNA: 6.

Focal cortical dysplasia (FCD) is a congenital abnormality of brain development in which neurons in an area of the brain fail to migrate properly.^37^ Interestingly, FCD is often diagnosed in people with PCDH19-CE, epilepsy or autism spectrum disorder (ASD).^9–11,38^ Furthermore, approximately 32% of people with PCDH19-CE fulfill the criteria for ASD, which includes comorbidities such as sensory alterations.^39^ Recently, we demonstrated that downregulation in the upper layer of the SSc of another cell-adhesion molecule (Negr1)—with a similar pattern of expression as PCDH19 and associated with human ASD—resulted in migration defects and core symptoms related to ASD (along with sensory alterations) in rodents.^40^ Thus, we investigated whether PCDH19 downregulation by a shRNA strategy^36^ in the SSc also resulted in migration defects and ASD-related behaviours. We used IUE at E17.5 to express efficient *Pcdh19* shRNAs^36^ or a control scramble vector in a subpopulation of neural progenitors that would normally migrate to Layer II/III of the SSc.^27,28,41^ Together with *Pcdh19* shRNA or control vector, we also electroporated a plasmid encoding for enhanced GFP- or Td-Tomato-fluorescent reporter proteins for better visualization of transfected neurons. In electroporated animals, we examined coronal sections of the SSc. We found that focal downregulation of PCDH19 impaired neuronal migration *in vivo* at P9, with no change in the layer identity. This was quantified as the number of migrating cells positioned in ectopic locations compared with controls, and layer-specific stainings (Fig. 3A and B; Supplementary Fig. 2A–C). Interestingly, the migration defect was maintained in early adulthood at P25 (Fig. 3C and D). Finally, since people with ASD may display abnormal cortical laminar organization accompanied by alterations in dendritic spines,^38,40,42–44^ we investigated the number of spines in neurons electroporated with *Pcdh19* shRNA versus control littermates. In line with previous literature on Layer V neurons,^23^ we found that Pcdh19*-*deficient neurons located in Layers II/III showed a similar number of spines per micrometer in comparison with controls (Supplementary Fig. 2D and E).

**Figure 4:**
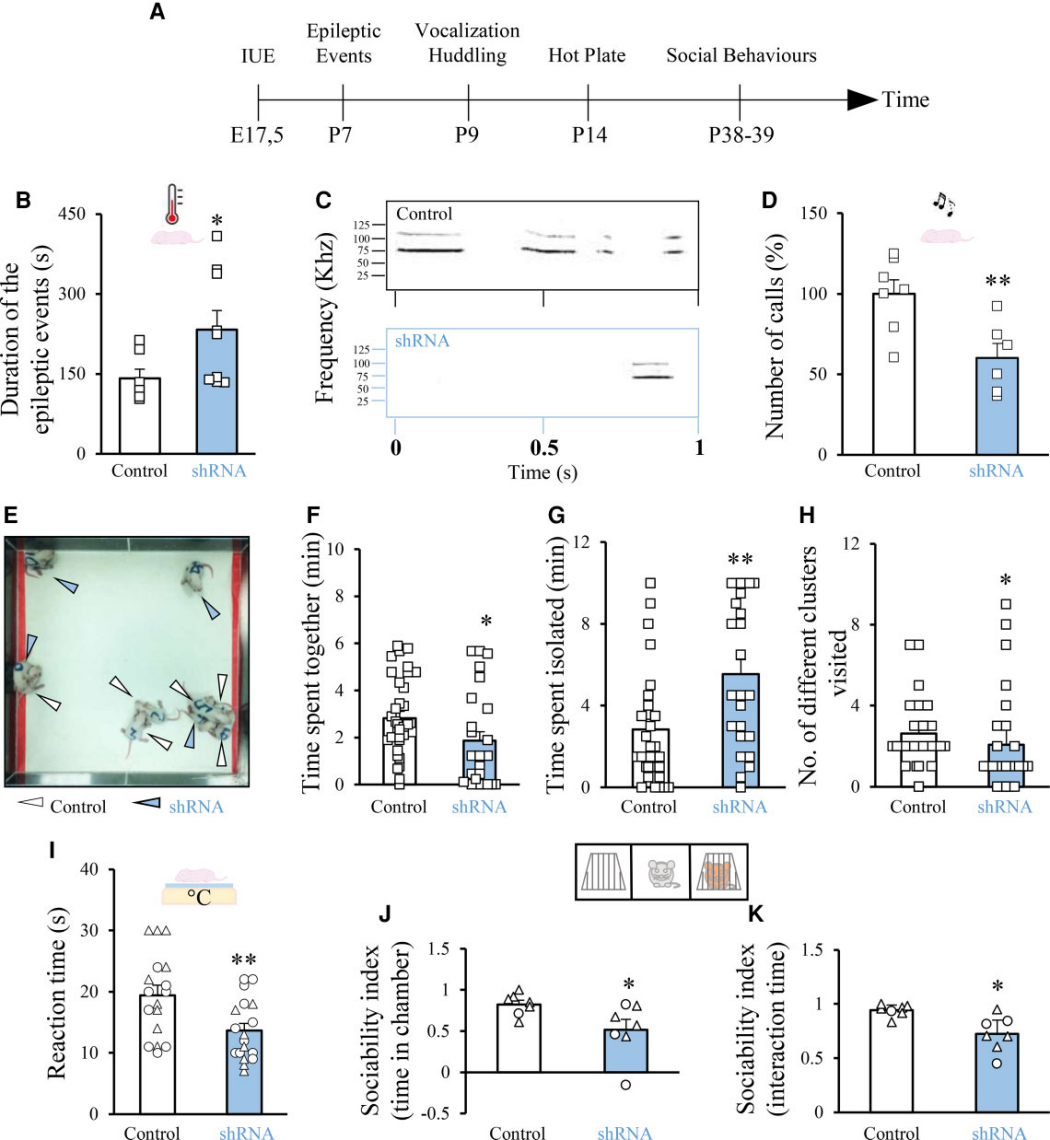
**Mosaic PCDH19 downregulation in the SSc causes heat-induced epilepsy and core/comorbid behaviours related to autism in rats**. (**A**) Schematic timeline of the behavioural tests. (**B**) Quantification of the total duration of epileptic events of pups electroporated *in utero* with either control vector or *Pcdh19* shRNA and subjected to an increased body temperature by exposure to a heating lamp at P7. Squares indicate values from single animals, and their averages (±SEM) are reported by bars. Mann–Whitney test: *P = 0.020. (**C**) Representative example of the USV spectrograms in P9 pups electroporated *in utero* with the *Pcdh19* shRNA or control vector, when isolated from their dam. (**D**) Quantification of the average number (±SEM) of ultrasound vocalizations of *Pcdh19* shRNA- or control vector-transfected pups expressed as percentage of the values of their control littermates, in experiments as in (**C**). Squares indicate values from single animals, and their averages (±SEM) are reported by the bars. Student’s *t*-test, **P = 0.009. (**E**) Representative screenshot of the huddling test at Minute 5, highlighting P9 pups electroporated *in utero* with a control vector or with *Pcdh19* shRNA. (**F, G, H**) Quantification of the Time Spent Together, Time Spent Isolated, and Number of Different Clusters Visited in huddling experiments as in (**E**). Mann–Whitney test, (**J**): *P = 0.02; (**L**): **P = 0.004, (**K**): *P = 0.02. Squares indicate values from single animals, and their averages (±SEM) are reported by bars. (**I**) Quantification of the paw withdrawal latency of P14-transfected pups after placement on a hot plate. Data are presented as the average time spent on the hot plate until the first pain reaction (±SEM). Circles indicate values from single female animals, triangles indicate values from single male animals, and averages for female and male animals together (±SEM) are reported by bars. Student’s *t*-test, **P = 0.008. (**J**) Sociability index based on the time spent inside the different chambers in the three-chamber sociability assay for P38–39 animals transfected *in utero* with a control vector or with *Pcdh19* shRNA. Circles indicate values from single female animals, triangles indicate values for single male animals, and total averages for female and male animals together (±SEM) are reported by bars. Mann–Whitney test, *P = 0.03. (**K**) Sociability index based on interaction time in the three-chamber sociability test. Circles indicate values from single female animals, triangles indicate values for single male animals, and total average for female and male animals together (±SEM) are reported by bars. Welch’s *t*-test, *P = 0.01.

The most debilitating core symptoms of people with PCDH19-CE are frequent (often fever-associated) epileptic events, appearing in early childhood (6–36 months; Depienne *et al*.;^1^ Specchio *et al*.;^57^ Depienne and Leguern;^2^ Trivisano *et al*.;^18^ Kolc *et al*.^6^). Thus, on a set of experimental animals similar to that which we used for the histological studies, we performed heat-induced epileptic seizures before sacrifice.^30^ In particular, we exposed control shRNA-transfected animals and *Pchd19* shRNA-transfected littermates at P7 to increasing hyperthermia by a heat lamp until reaching 39–41.5°C for 20 min. Pups electroporated with *Pcdh19* shRNA experienced a significantly higher duration of epileptic events (spasms, tonic-clonic, and stretching) than their control littermates, with only a not significantly higher number of epileptic events [during the entire duration of the test (Fig. 4A and B; number of events not shown: control: 7.2 ± 0.5 total number of events per minute; *Pcdh19* shRNA: 8.5 ± 0.7 total number of events per minute)].

PCDH19-CE people also present impaired social behaviour related to ASD.^3,39^ USV is a commonly used behavioural test for social/communication deficits in ASD animal models.^45^ Pup littermates electroporated with either control vector or *Pcdh19* shRNA were separated from their mother and littermates and kept in isolation for 5 min at P9. During that time, we recorded USVs and then calculated their frequency. We found that *Pcdh19* shRNA-transfected pups vocalized significantly less than their control littermates (Fig. 4C and D). Next, in another cohort of control and *Pcdh19* shRNA-electroporated pups, we analyzed huddling behaviour, which is also considered a social behaviour in rodents.^31,46^ To this aim, each litter (of five pups previously electroporated with *Pchd19* shRNA and five pups electroporated with control construct) was separated from their mother and placed in an empty area, where interactions among pups were video-recorded (Fig. 4E).^31^ In agreement with reduced social behaviours as measured by USVs, pups transfected with *Pcdh19* shRNA spent less time together (Fig. 4F), more time isolated from other animals (Fig. 4G) and visited a decreased number of diverse clusters (Fig. 4H), in comparison with their control littermates. No significant difference was observed in terms of cluster switches (not shown, control: 1.60 ± 0.60, Pcdh19: 1.00 ± 0.00). Furthermore, we also examined whether PCDH19 downregulation in the SSc caused sensory alterations, including increased pain sensitivity, as already described in people with PCDH19-CE^39,47^ and as a comorbidity of ASD.^48^ At P14, we observed that pups transfected with *Pcdh19* shRNA presented a significantly shorter latency to respond to an acute thermal stimulus than control pups, when placed on a heated plate (Fig. 4I).

Next, analogous to our study on impaired cortical migration, we tested whether the social behaviour impairment of *Pchd19* shRNA-transfected animals persisted into adulthood (P38–39) by performing a classical behavioural paradigm to assess social behaviour in ASD mouse models (i.e. the three-chamber test; Eissa *et al*.)^35^. In particular, we evaluated the social interactions of *Pcdh19* shRNA animals or control animals upon exposure to a novel rat of the same sex (Stimulus 1) versus an object (expressed as sociability index) and to a novel rat (Stimulus 2) versus previously met Stimulus 1 (expressed as social novelty index). We found that *Pcdh19* shRNA showed a significantly decreased ‘Sociability Index’ (Fig. 4J and K) and a non-significant tendency toward a decreased ‘Social Novelty’ index [data not shown; Discrimination Index (time in the chamber): control: 22.49 ± 9.87%, Pcdh19 shRNA: −4.19 ± 10.84%; Discrimination Index (interaction time): control: 23.42 ± 13.36%, Pcdh19 shRNA: 27.52 ± 14.35%], in comparison with the control rats. Altogether, these data demonstrate that PCDH19 in the cortex regulates neuronal migration and core behaviours relevant to PCDH19-CE, such as heat-induced epilepsy and ASD.

### PCDH19 downregulation in the hippocampus affects structural layering and impairs cognitive function

**Figure 5:**
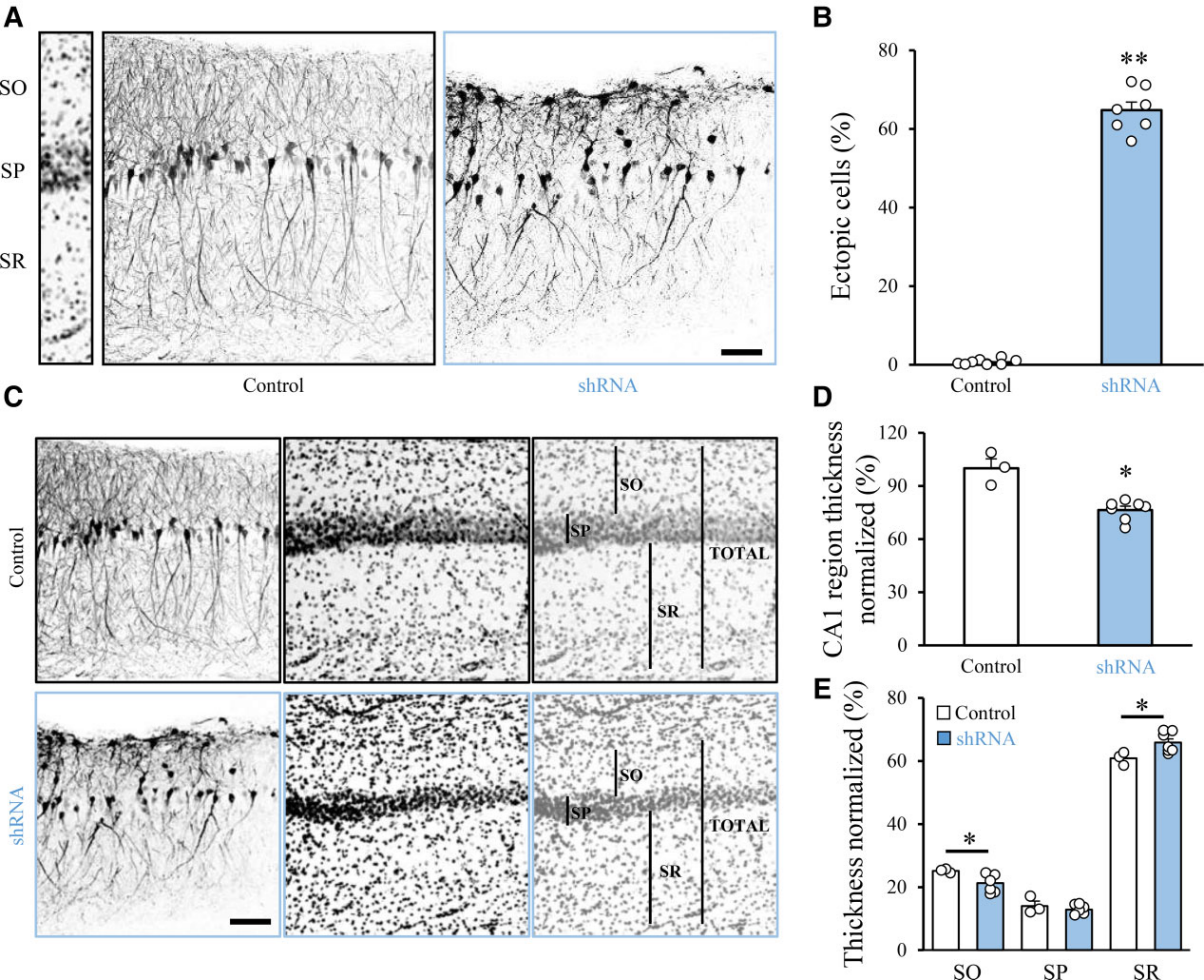
**Mosaic PCDH19 downregulation in the hippocampus impairs neuronal migration and sublayer thickness**. (**A**) Confocal images of GFP fluorescence in coronal sections of the rat hippocampus at P25 after *in utero* transfection at E17.5 with control vector or *Pcdh19* shRNA. Slices were counterstained with nuclear staining Hoechst, for visualization of hippocampal layers (left). SO = stratum oriens, SP = stratum pyramidale, SR = stratum radiatum. Scale bar: 50 μm. (**B**) Quantification of the number of ectopic cells transfected with either control vector or shRNA against *Pcdh19*. Numbers are expressed as a percentage of the ectopic cells normalized to the total number of fluorescent cells in the same section (± SEM). Mann–Whitney test: **P = 0.001. Total number of animals processed (one slice/animal). (**C**) Confocal images of GFP fluorescence in coronal sections of the rat hippocampus at P25 after IUE. Slices were counterstained with Hoechst. Black bars show thicknesses of indicated layers. Scale bar: 50 μm. (**D, E**) Quantification of the thickness of the total CA1 region (**D**) or its subregions (**E**) in coronal slices transfected as in (**C**). Numbers are expressed as a percentage normalized to the controls (± SEM); Number in parentheses: total number of animals processed (one slice/animal). (E) Student’s *t*-test: *P = 0.03; (**F**) (SO) Welch’s *t*-test: *P = 0.01, (SP) Welch’s *t*-test: n.s., (SR) Welch’s *t*-test: *P = 0.03. Total number of animals processed (one slice/animal). Number of data points used for the graph: control: 3, *Pcdh19* shRNA: 3.

PCDH19 is highly expressed in the hippocampus (Fig. 1F), and its downregulation impairs the migration and morphology of hippocampal pyramidal neurons at P7 *in vivo.*^36^ To investigate whether the migration defect caused by PCDH19 downregulation in the hippocampus was also persistent later in life, we electroporated littermate rat embryos at E17.5 with a control vector or *Pcdh19* shRNA using tripolar IUE.^27,28^ In hippocampal brain slices, we quantified the number of transfected cells located in the stratum oriens (SO) and SP at P25. While neurons transfected with the control vector were aligned along the SP, a number of *Pcdh19* shRNA-expressing neurons remained ectopically located in the SO (Fig. 5A and B). Furthermore, in *Pcdh19* shRNA-transfected rats, ectopic neurons were accompanied by a reduction in the general thickness of the CA1 region of the hippocampus (Fig. 5C and D). Moreover, hippocampal sublayers also showed thickness differences between control and treated animals (Fig. 5C and E). Next, analogous to our analysis in cortical cells, we also performed dendritic spine counting in hippocampal slices. In hippocampal neurons transfected with *Pcdh19* shRNA, we found a similar spine density compared with controls (Supplementary Fig. 2F and G).

**Figure 6:**
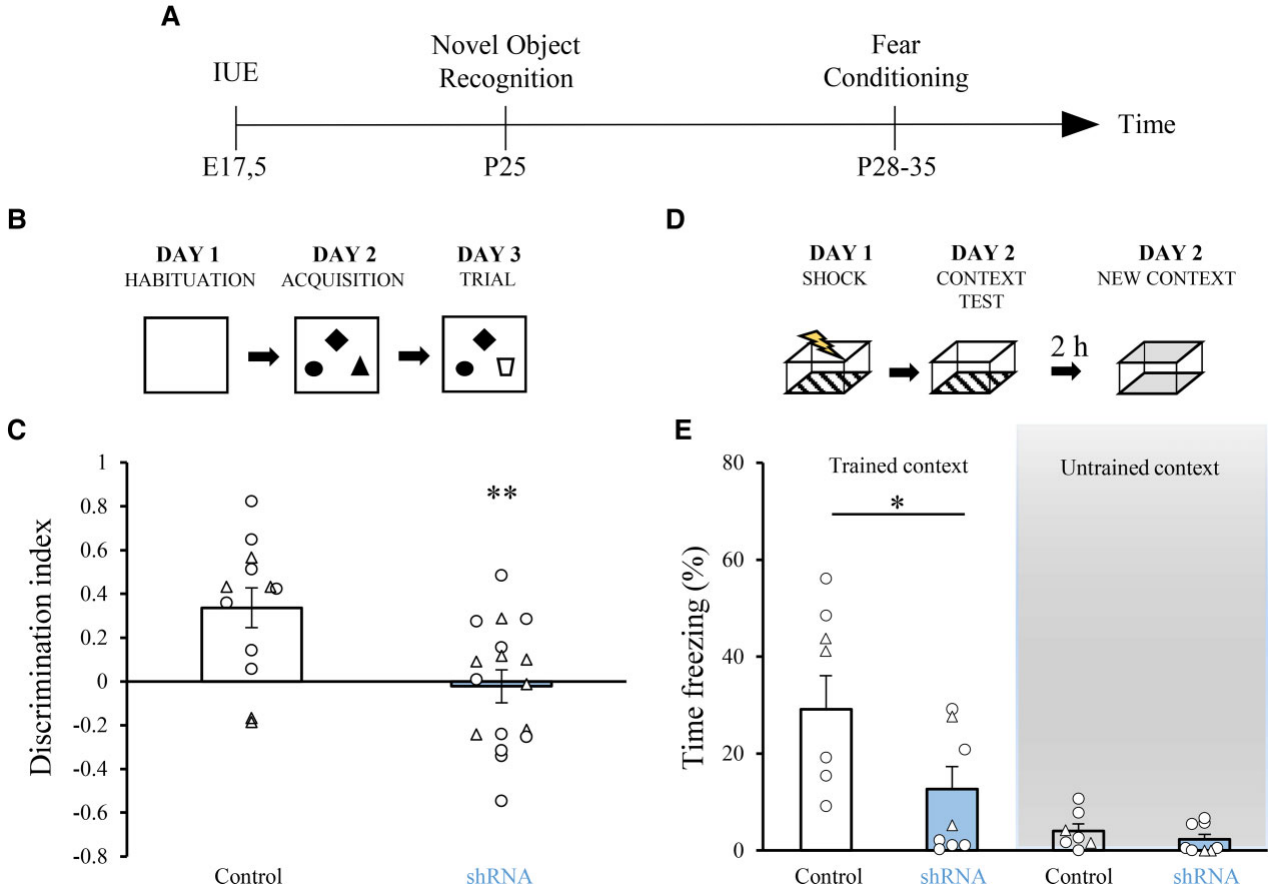
**Mosaic PCDH19 downregulation in the hippocampus leads to cognitive impairments**. (**A**) Schematic timeline of the behavioural tests. (**B**) Experimental protocol for the NOR test. (**C**) Quantification of the discrimination index in P25 animals transfected *in utero* with control or *Pcdh19* shRNA. Circles indicate values from single female animals, triangles indicate values from single male animals, and averages for female and male animals together (±SEM) are reported by bars. Student’s *t*-test: **P = 0.05. (**D**) Experimental protocol for the CFC test. (**E**) Quantification of the freezing response in 4- to 5-week-old transfected animals upon exposure to a trained (left) or untrained (right) context. Circles indicate values from single female animals, triangles indicate values from single male animals, and averages for female and male animals together (±SEM) are reported by bars. Welch’s *t*-test *P = 0.02, left; and Mann–Whitney test: *P* = not significant, right.

Cognitive impairment ranging from mild to severe is one of the core symptoms of PCDH19-CE.^3,5,2,49,50^ Thus, we hypothesized that a defective development of the hippocampus (as one of the main brain regions involved in learning and memory^51^) by PCDH19 downregulation may contribute to the cognitive impairment present in PCDH19-CE. To test this hypothesis, we assessed control or *Pcdh19* shRNA animals in two independent behavioural tests related to hippocampus-dependent cognitive functions. In particular, we first evaluated long-term explicit memory in the NOR (Fig. 6A and B) at 4–5 weeks of age in animals electroporated *in utero*. Electroporated *Pcdh19* shRNA animals showed poor novelty-discrimination capability between a familiar object and a novel object (Fig. 6C). Additionally, no significant differences were observed in total object exploration or object preference [data not shown; total exploration time (acquisition): control: 57.48 ± 6.7 s, *Pcdh19* shRNA: 91.48 ± 8 s; total exploration time (trial): control: 47.44 ± 5.2 s, *Pcdh19* shRNA: 48.31 ± 6.44 s; object preference for control: Object A: 39.25 ± 2.69%, Object B: 30.13 ± 2.97, Object C: 30.60 ± 2.73%; Object preference for *Pcdh19* shRNA: Object A: 32.62 ± 3.16%, Object B: 38.88 ± 4.62%, Object C: 28.49 ± 3.79%], indicating that the poor performance in the NOR was not due to alterations in total object exploration or object preference. Next, we tested the same control and *Pcdh19* shRNA animals for associative memory in the contextual fear-conditioning test (CFC; Fig. 6A and D). We found that animals transfected with *Pcdh19* shRNA showed significantly impaired memory in the CFC test, as demonstrated by a strong reduction of a freezing response elicited upon re-exposure to a training context 24 h after conditioning with an electric shock (Fig. 6E). *Pcdh19* shRNA animals showed a similar freezing behaviour in comparison with control animals in an untrained context (Fig. 6E).

Altogether, these results indicate that PCDH19 downregulation in the hippocampus is associated with hippocampal structural malformation and impairment in cognitive functions relevant to PCDH19-CE.

## Discussion

Using IUE to achieve a focal mosaic of WT and PCDH19-deficient cells, we demonstrated here that PCDH19 in the rat cortex and hippocampus is required for proper neuronal migration, seizure susceptibility, core/comorbid behaviours related to ASD and cognitive functions, which are altogether consistent with the PCDH19-CE phenotype in people.

The *PCDH19* gene is localized on chromosome X. As a result of random X-chromosome inactivation, PCDH19 has a mosaic pattern of expression in females.^2^ In this context, we proposed a quick, easy, and useful approach to mimic the mosaic pattern of expression of PCDH19 using IUE. In particular, we used this technique to achieve focal transfection of only a certain number of progenitors in the electroporation region and generate a mosaic of PCDH19-downregulated and WT cells. Interestingly, while FCD, ectopic neurons in the cortex, and hippocampal sclerosis were reported in PCDH19-CE people,^7,10,11^ no major brain developmental abnormalities (apart from abnormal cell segregation, subtle changes in cortical layer composition and mossy fibre presynaptic development) have been clearly detectable and/or carefully observed in *Pcdh19* knockout or heterozygous mice^8,11,21,22,23,25,26^ (Table 3). Moreover, neuronal migration upon PCDH19 downregulation has not been specifically addressed in other PCDH19-CE mouse models^8,11,21–23^ (Table 3). Here, in electroporated rats, we found a significant migration delay and detected ectopic neurons at P9 that persisted later in adulthood, which was due to a downregulation of PCDH19 in the subpopulation of neural progenitors destined to the upper layers of the developing cortex or progenitors of principal neurons of the developing hippocampus. This is in line with motility studies on *Pcdh19* null neurons *in vitro*^21^ and other studies *in vivo*, where loss of PCDH19 expression disrupted migration in the rat hippocampal formation^25,36^ or during neurulation in zebrafish.^52^ Furthermore, the high number of *Pcdh19* shRNA cells found in proximity of the ventricular zone at P9 may also suggest alterations in the cell cycle of the transfected progenitors of the somatosensory. The close relation between cell adhesion and cell cycle phase progression in our experimental setting will require further investigation.^53^ This is even more relevant in light of the proven role of N-cadherin in the regulation of neuronal stem cell quiescence,^20^ since N-cadherin is a well-known partner of PCDH19 in adhesion,^54^ even in the context of PCDH19-CE.^25^

**Table 3:** Summary of the available animal models for PCDH19-CE by *Pcdh19* expression reduction

Species	Genetic manipulation/Strategy		Brain malformations		Behavioural deficits		Reference
	Female	Male	Female	Male	Female	Male	
Mouse	Heterozygous *(Pcdh19^+/–^)*	Pcdh19 KO *(Pcdh19^-/y^)*	Subtle changes in cortical layer composition		Reduced anxiety (Confirmed with elevated plus maze) Increased exploratory behaviour (Confirmed by open field test)		^26^
Mouse	Heterozygous *(Pcdh19^+/–^)*	Hemizygous *(Pcdh19^-/y^)*	Impaired mossy fibre presynaptic development and function	No change	Defects in cognitive behaviours (Confirmed by contextual discrimination fear conditioning test)	No change	^25^
Mouse	Heterozygous *(Pcdh19^+/–^)* Knock out *(Pcdh19*^–/–^*)*	Hemizygous *(Pcdh19^-/y^)*	NA	NA	Lower seizure thresholds and qualitatively more severe seizure phenotypes (confirmed by 6-Hz psychomotor test and flurothyl injection)	No change	^24^
Mouse	Heterozygous (*Pcdh19* ^XLacZ/X^)	Pcdh19 KO (*Pcdh19* ^XLacZ/Y^)	Striped pattern of segregation between PCDH19(+) and PCDH19(−) cells in the brain due to abnormal cell segregation in the cortex	No visible segregation defect	Autism-like behaviour (confirmed by three-chamber test)	Autism-like behaviour (confirmed by three-chamber test, reciprocal social interaction test, rearing, self-grooming)	^22^
Mouse	*Pcdh19^HA-FLAG/β-Geo^*	NA	Striped pattern of segregation between PCDH19(+) and PCDH19(−) cells in the brain due to abnormal cell segregation in the cortex	NA	Altered network brain activity (confirmed by electrocorticogram)	NA	^11^
Rat	*In utero* electroporation of *Pcdh19* shRNA		Impaired migration, orientation and dendritic arborization of CA1 hippocampal neurons		Increased seizure susceptibility (Confirmed by PTZ injections)		^36^
Mouse	*In utero* electroporation Mix of pNeuroD-Cre, pCAG-loxP-neo-pA-loxP-GFP and pCALNL5-Pcdh19-FLAG		Clustering of neurons in the cerebral cortex		NA		^23^
	Heterozygous (*Pcdh19X^+^/X^LacZ^*)	NA	Patchy distribution of the cells in the cortex	NA	NA	NA	
	Heterozygous (*Pcdh19X^+^/X^−^*)	Hemizygous (*Pcdh19X^−^/Y*)	NA	NA	Anxiety-like behaviour (confirmed by light/dark transition, elevated plus maze) Abnormal mobility under stress conditions (confirmed by tail suspension test, Porsolt forced swim test, open field) Decreased fear responses (confirmed by fear-conditioning test)	Anxiety-like behaviour (confirmed by elevated plus maze) Abnormal mobility under stress conditions (confirmed by tail suspension test, Porsolt forced swim test, open field) Sensory perception (confirmed by hot plate)	
	Homozygous (*Pcdh19*X^−^/X^−^)		NA		NA		
Mouse	*Pcdh19^+/β-Geo^*	*Pcdh19^Y/β-Geo^*	No gross defects in brain morphology	No gross defects in brain morphology	Healthy and fertile	Healthy and fertile	^21^

NA: data not available.

The mechanisms by which PCDH19 regulates neuronal migration are still unknown. However, as for other CAMs,^55^ it is possible that the observed migration impairment may be caused by loss, incorrect expression or simply misfunction of the extracellular domain of PCDH19 on the surface of migrating neurons, resulting in miscommunication with other cells/adhesion molecules. For example, one of the possible hypothesis for these defective molecular interactions points again to mismatched interactions between PCDH19 and N-cadherin in *Pcdh19^+/–^* mice.^25^ This would affect signalling and, in turn, presynaptic development.^25^ Interestingly, most of the mutations of *PCDH19* in PCDH19-CE people are related to extracellular domains responsible for the interactions with partner cells.^56^

In our PCDH19-CE rat model, together with the deficits in brain development, we also described a number of behavioural phenotypes related to core and comorbid symptoms of people with PCDH19-CE. Among the known rodent (i.e. mouse) models for PCDH19 dysregulation that reported milder brain developmental phenotypes, only some displayed also behavioural phenotypes (i.e. ASD-related behaviours in *Pcdh19X^LacZ^/X*, *Pcdh19X^+^/X* and *Pcdh19X^−^/Y* mice^22,23^ cognitive impairments in *Pcdh19*^+/−^ females, but not *Pcdh19*^−/y^ male mice^25^) reduced anxiety and increased exploratory behaviour both in *Pcdh19X^+^/X* and *Pcdh19X^−^/Y*,^26^ whereas in some others, behaviours were not investigated (i.e. *Pcdh19^HA-FLAG^/^β-Geo^* and *Pcdh19^+/β-Geo^*^11,21^  Table 3). Conversely, some other models (i.e. *Pcdh19X^LacZ^/Y*^22^) did not show (or were not visualized) gross brain developmental abnormalities, but they did show some ASD-related behavioural phenotypes. Finally, a recent study reported increased 6-Hz psychomotor- or pharmacological-induced seizures in female mice (*Pcdh19*^+/−^ and *Pcdh19*^–/–^ Rakotomamonjy *et al*.)^24^. Nevertheless, males (*Pcdh19*^−*/y*^) animals remained seizure-free, and brain developmental phenotypes or ASD-related behaviours were not investigated (Table 3). We found here that in rats electroporated with *Pcdh19* shRNA in the SSc or hippocampus, brain developmental abnormalities were accompanied by a number of aberrant behaviours. In particular, SSc *Pcdh19* shRNA-electroporated rats showed increased heat-induced seizures. Notably, leading clinical features of PCDH19-CE people point to early onset of seizures accompanied by fever episodes as one of the characteristics of the condition.^6,18,57^ Thus, the results on the SSc obtained here complement (with a more disease-relevant seizure induction protocol) previous results on increased susceptibility to seizures in rats electroporated with *Pcdh19* shRNA in the hippocampus by injection with the γ-aminobutyric acid type A (GABA_A_) receptor antagonist pentylenetetrazol (PTZ)^36^ or in female *Pcdh19^+/−^* and *Pcdh19*^–/–^ mice by flurothyl injection or 6-Hz psychomotor test.^24^

A large number (32%) of people with PCDH19-CE also carry a diagnosis of ASD^39,48^ with social deficits early in life that persist and may become more prominent in adulthood.^1^ Here, in animals transfected with *Pcdh19* shRNA in the SSc, we found impaired social behaviours in terms of decreased vocalization and huddling behaviour in young pups and poor sociability in adult animals.

Formal diagnosis of ASD in people with PCDH19-CE also includes aberrant sensory perception, with both hypo- or hypersensitivity to pain reported as a comorbidity.^39,47,48^ Here, we found that pups with PCDH19 downregulation in the SSc showed faster paw withdrawal (thus, hypersensitivity) than their control littermates in the hot plate test. In particular, the effect seemed higher in males than in females (females; control: 16.75 ± 1.9 s, *Pcdh19* shRNA: 14.81 ± 1.54 s; males; control: 21.77 ± 2.41 s, *Pcdh19* shRNA: 11.85 ± 1.63 s). This is in agreement with the only mouse model of PCDH19-CE (i.e. *Pcdh19X^−^/X^+^* and *Pcdh19X^−^/Y*) where pain sensitivity was tested, and where only males showed decreased pain tolerance^23^ (Table 3). Thus, both this latter study and ours suggest that certain phenotypes are stronger in females versus males in animal models. Interestingly, the recently discovered complex PCDH19-NONO has been identified as a positive co-regulator of oestrogen receptor alpha (ERα)-mediated gene expression (Pham *et al*., 2017). Moreover, expression of the PCDH19-mutant form affected a subset of known ERα-regulated genes (Pham *et al*., 2017). Altogether, this points to possible hormone-related effects that may contribute (together with cell mosaicism) to stronger or opposite behavioural phenotypes in PCDH19-CE females versus males.

Finally, although cognitive dysfunction is one of the core symptoms of PCDH19-CE,^2,3,5,49,50^ only two animal models [i.e. *Pcdh19* heterozygous females (*Pcdh19X^+^/X^−^*) and (*Pcdh19^+/–^*)] showed decreased responses in the fear conditioning test,^23,25^ whereas hemizygous males [i.e. (*Pcdh19X^−^/Y*) and (*Pcdh19^-/y^*)] did not show alterations,^23,25^ and the other models have not been tested^8,11,21,22,24^ (Table 3). Here, we found that rats with PCDH19 downregulation in the hippocampus subjected to two independent cognitive tests showed a significant decrease in long-term cognitive functions, which was also described in PCDH19-CE people.^1,2,58,59,60^

Thus, PCDH19-CE is a complex disease characterized by a number of behavioural symptoms. To the growing arsenal of animal models of PCDH19-CE that have proven efficient in recapitulating either brain abnormalities or behavioural phenotypes, we add a rat model of mosaic PCDH19 downregulation, which has been developed by using IUE and proved useful for studies on PCDH19-dependent brain development and PCDH19-CE-related behaviours. This model demonstrated to be technically easy, very versatile, quick and economical to set up.^27,28,41^ Notably, our animal model, in comparison with the other PCDH19-CE rodent models, allows selective transfection of specific brain regions of choice (i.e. here SSc or hippocampus). This is fundamental to clearly dissect the contribution of PCDH19 expressed in a specific brain region to selected behaviours. Potentially, other brain regions could be easily targeted with *Pcdh19* shRNA by IUE,^27,28^ further increasing the versatility and potential of our approach and rat model of PCDH19-CE. Moreover, since our model presents both developmental brain abnormalities and behavioural phenotypes related to PCDH19-CE, it may be useful to test new therapeutic approaches aimed at rescuing the brain developmental trajectory and/or related behavioural phenotype in PCDH19-CE. For example, the modulator of GABA_A_ receptor activity Ganaxalone (GNX) is currently in a Phase 2 clinical trial for PCDH19-CE (Clinical Trials Identifier: NCT03865732), but it has never been tested in animal models of PCDH19-CE. This is possibly due to the lack of a reliable and disease-relevant seizure phenotype in previous mouse models. Our rat model may be used to help test GNX for improvement in seizure susceptibility in the PCDH19-CE-relevant experimental paradigm of heat-induced seizure. Furthermore, given the fundamental role that GABA_A_ signalling exerts in brain development^61^ and ASD-related behaviours,^62^ including pain sensitivity^39,48^ and regulation of cognition,^63^ our rat model may be used to help test the ability of GNX to rescue brain morphological maturation (when administered to pups) and/or also the other accompanying PCDH19-CE-related behavioural phenotypes.

In conclusion, we present a new, versatile animal model for easy, efficient, and detailed studies on the mechanism underlying brain development and behavioural abnormalities due to PCDH19 deficiency using cellular interference *in vivo*. In addition, this animal model will possibly help researchers design and/or preclinically validate future therapeutic approaches to rescue seizures, core and comorbid behaviours related to ASD, and cognitive impairment in PCDH19-CE people.

## Supplementary Material

fcac091_Supplementary_Data

## Abbreviations

ASD =: autism spectrum disorder
CFC =: contextual fear conditioning
E =: embryonic
FCD =: focal cortical dysplasia
GFP =: green fluorescent protein
GABA_A_ =: γ-aminobutyric acid type A
GNX =: Ganaxalone
IUE =: *in utero* electroporation
NGS =: normal goat serum
NOR =: novel object recognition
P =: postnatal
PBS =: phosphate-buffered saline
PCDH19 =: protocadherin 19
PCDH19-CE =: PCDH19 clustering epilepsy
PTZ =: pentylenetetrazol
shRNA =: short hairpin RNA
SO =: stratum oriens
SSc =: somatosensory cortex
SP =: stratum pyramidale
USV =: ultrasonic vocalization
WT =: wild-type
