# Supplementary material for: A rat model of a focal mosaic expression of PCDH19 replicates human brain developmental abnormalities and behaviours
Source: Brain Commun. 2022 Apr 5;4(3):fcac091. doi: 10.1093/braincomms/fcac091 (PMC9070467; doi:10.1093/braincomms/fcac091)
Supplement: fcac091_Supplementary_Data [file fcac091_supplementary_data.pdf]

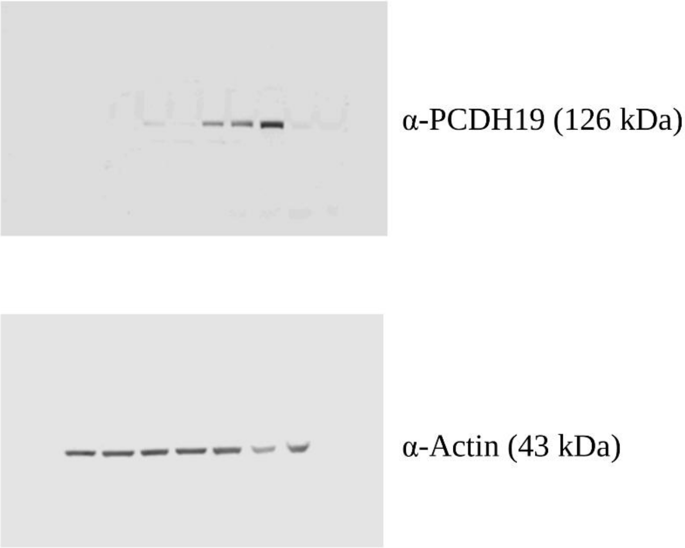

Supplementary figure 1. Full- size and uncropped blots used for Main Figure 1A.

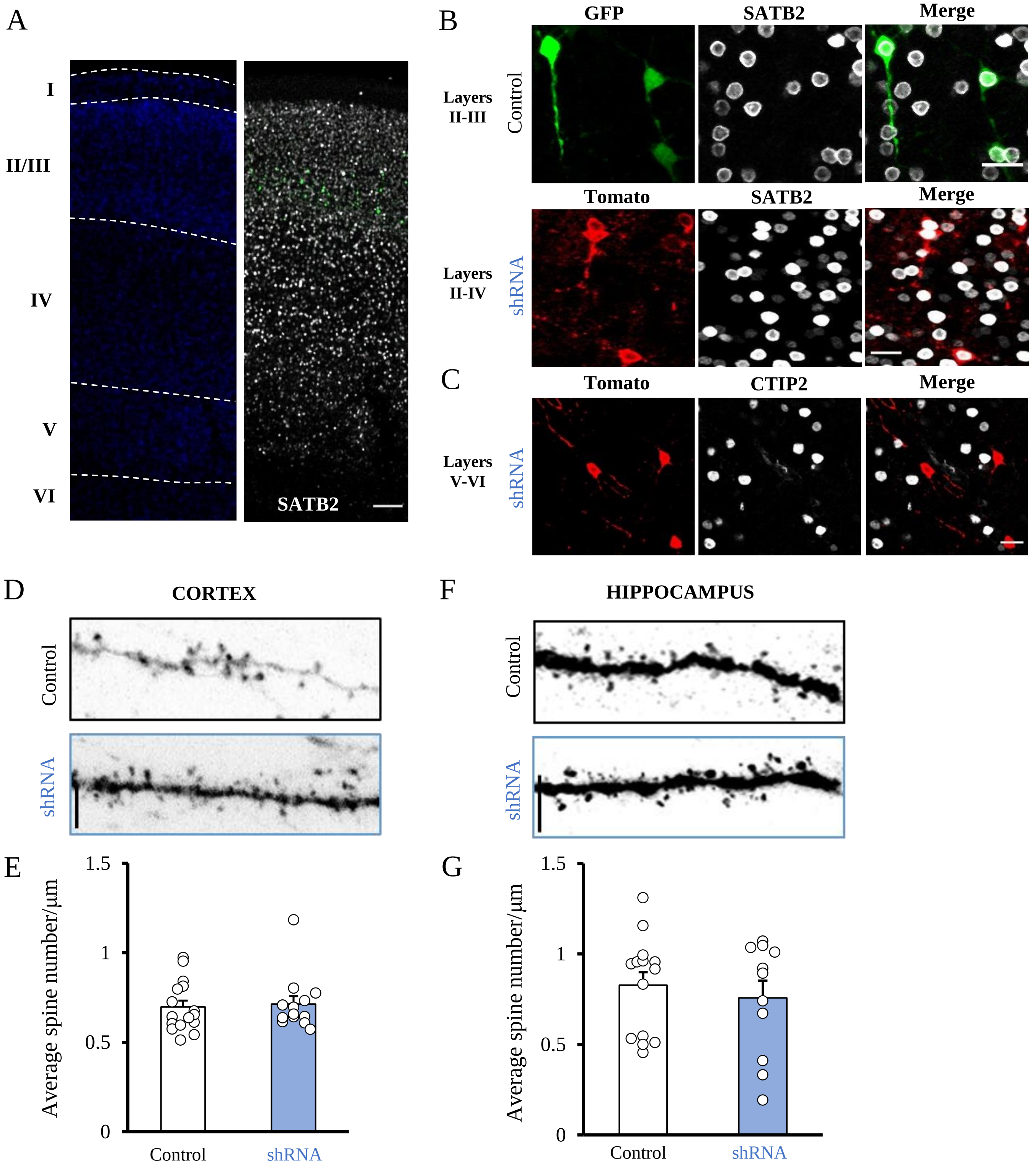

Supplementary figure 2. Mosaic PCDH19 downregulation of upper layer neurons in the somatosensory cortex does not change their layer identity and dendritic spine number.

(A) Representative fluorescence images of somatosensory-cortex slices from animals transfected *in utero* at E17.5, and stained at P8 for upper-layer marker SATB2 (white, right), and counterstained with DAPI (blue, left). Scale bar, 100 µm. **(B)** Representative fluorescence magnification images of neurons located at layers II/IV from experiments as in A. GFP (green) is the reporter that was used for control shRNA. Tomato (red) is the reporter that was used for PCDH19 shRNA. Scale bars, 20 µm. **(C)** Representative fluorescence magnification images of neurons located at layers V/VI from experiments as in A. Slices were stained for deep-layer marker CTIP2 (white). Scale bar, 20 µm. **(D)** High magnification confocal images of GFP fluorescence in neurons from the rat cortex at P35 after *in utero* transfection (at E17.5) with Control vector or Pcdh19 shRNA. Scale bar, 5µm. **(E)** Quantification of cortical dendritic spine number in experiments as in D. Lines report the average spine density (± SEM) and circles represent data points for each neuron. Unpaired two-tailed Student's t test (*p < 0.05, ***p<0.001). **(F)** High magnification confocal images of GFP fluorescence in neurons from the rat hippocampus at P25 after *in utero* transfection (at E17,5) with Control vector or Pcdh19 shRNA. Scale bar, 5µm. **(G)** Quantification of cortical dendritic spine number in experiments as in F. Lines report the average spine density (± SEM) and circles represent data points for each neuron. Unpaired two-tailed Student's t test (*p < 0.05, ***p<0.001)
